# COL1A2 and APOLD1 Define a Dual-Axis Molecular Framework for Diabetic Nephropathy-Retinopathy Comorbidity: An Integrative Multiomics and Machine Learning Study in Mouse Models

**DOI:** 10.7150/ijms.136651

**Published:** 2026-08-11

**Authors:** Jinyu Li, Jingliang He, Yirui Zhu, Jiajia Yuan, Zhiyong Zhang, Shuo Yang

**Affiliations:** 1 Zhejiang University, Eye Center of Second Affiliated Hospital, School of Medicine, China. Zhejiang Provincial Key Laboratory of Ophthalmology. Zhejiang Provincial Clinical Research Center for Eye Diseases. Zhejiang Provincial Engineering Institute on Eye Diseases, Hangzhou, China; 2 Cambridge Eye Unit, Addenbrooke's Hospital, Cambridge University Hospitals, Cambridge, UK; 3 Department of Ophthalmology, Renmin Hospital of Wuhan University, Wuhan University, Wuhan, China

**Keywords:** Diabetic Retinopathy, Diabetic Nephropathy, Comorbidity, COL1A2, APOLD1, Machine Learning, Biomarkers

## Abstract

**Background:**

Diabetic retinopathy (DR) and diabetic nephropathy (DN) are severe microvascular complications that frequently co-occur, suggesting shared pathogenic mechanisms. However, systematic identification of their common molecular drivers remains limited.

**Methods:**

We performed an integrative multiomics analysis combining bulk transcriptomics (six datasets: GSE30528, GSE30529, GSE96804, GSE142025, GSE160306, and GSE221521), single-cell RNA sequencing (scRNA-seq; GSE216510 for DN, GSE178121 for DR), advanced computational modeling, and experimental validation. Differential expression analysis, weighted gene coexpression network analysis (WGCNA), and protein-protein interaction (PPI) network analysis were conducted, and 120 combinatorial machine learning models were constructed. Cell-cell communication and pseudotime trajectory analyses were performed. Western blotting was used to validate protein expression dynamics in db/db (type 2) diabetic mouse models at 1, 3, and 6 months after diabetes onset.

**Results:**

Cross-tissue analysis revealed *COL1A2* as a consistently upregulated gene and *APOLD1* as a consistently downregulated gene in both DR and DN. These two genes define a dual-axis model: an early dysfunction axis marked by downregulated APOLD1 expression and a late structural remodeling axis driven by upregulated COL1A2 expression. Machine learning models built on comorbidity signatures achieved robust predictive performance in hold-out validation. scRNA-seq revealed that in DN, *COL1A2* and *APOLD1* were specifically expressed in fibroblasts; in DR, they were predominantly expressed in pericytes/vascular smooth muscle cells, with APOLD1 also detected in endothelial cells. Pseudotime analysis indicated that APOLD1 expression peaked early in disease trajectories, whereas COL1A2 expression accumulated at later stages. Western blotting confirmed the progressive upregulation of COL1A2 and downregulation of APOLD1 protein expression in both retinal and renal tissues over time in diabetic models, with consistent trends across the 1-, 3-, and 6-month timepoints.Cell-communication analysis revealed extensive network dysregulation: DN exhibited SPP1, RANKL, CD45, CSF, and FGF pathway activation and WNT, ARGN, and NOTCH pathway suppression, whereas DR exhibited MHC-I, EDN, LAMININ, and VEGF pathway suppression. *In silico* COL1A2 knockout in fibroblasts (DN) and smooth muscle cells (DR) induced distinct transcriptional responses-immune-related in DN versus vascular stress-related in DR.

**Conclusion:**

On the basis of the study results, we propose a novel “dual-axis” model for diabetic microvascular comorbidity: an early dysfunction axis marked by downregulated *APOLD1* expression and a late structural remodeling axis driven by upregulated *COL1A2* expression. These findings provide a cohesive molecular framework and potential biomarkers for the concurrent pathogenesis of DR and DN.

## Introduction

Diabetic retinopathy (DR) and diabetic nephropathy (DN) are the most common microvascular complications of diabetes, representing the leading causes of blindness and end-stage renal disease, respectively, in working-age populations and imposing a substantial burden on global health care systems 1, 2. Clinical epidemiological data indicate that approximately 30-40% of patients with DN also have DR, and vice versa, a phenomenon increasingly recognized as “renal-retinal syndrome” 3, 4. Although traditional risk factors such as hyperglycemia and hypertension are well recognized, the core molecular mechanisms driving this comorbidity remain largely elusive.

Previous studies have revealed several shared pathways involved in diabetic microvascular injury, including oxidative stress, renin-angiotensin system activation, and chronic low-grade inflammation 5-7. Endothelial dysfunction is a common central event in both DR and DN, as it involves the blood-retinal barrier and the glomerular filtration barrier, respectively 8, 9. In addition, the dysregulation of growth factor signaling, such as that involving VEGF and TGF-β, and pathological extracellular matrix accumulation are also shared features of both complications 10, 11. Genetic susceptibility and epigenetic regulation, including those by noncoding RNAs, have been implicated in disease progression 12, 13. However, most of the relevant studies have focused on single tissues or individual pathways; therefore, there is a lack of a systematic understanding of the molecular basis underlying DR-DN comorbidity. Moreover, there is an urgent clinical need for novel biomarkers that can predict comorbidity risk early, reflect disease progression, and guide intervention strategies.

In this study, we systematically dissected the core molecular signatures of DR and DN comorbidity by integrating multiomics data and machine learning approaches. We combined multiple bulk transcriptome dataset analyses with weighted gene coexpression network analysis to identify disease-associated modules, screened the most predictive feature genes through 120 combinatorial machine learning models, resolved the cell type-specific expression patterns of key genes using single-cell RNA sequencing, reconstructed disease progression trajectories through pseudotime analysis, and delineated alterations in the microenvironmental signaling network via cell-communication analysis. Finally, we validated, via Western blotting, the dynamic changes in the protein levels of the core biomarkers in retinal and renal tissues from diabetic animal models. The aims of this integrative strategy were to transcend traditional differential gene lists, identify and validate the core molecular drivers of DR-DN comorbidity, and provide new targets for early warning, risk stratification, and targeted intervention in renal-retinal syndrome.

## Research Design and Methods

### Bulk Transcriptome Data Acquisition and Processing

Six public datasets were downloaded from the Gene Expression Omnibus (GEO) database: GSE30528, GSE30529, GSE96804, GSE142025 (DN studies), GSE160306, and GSE221521 (DR studies). The GSE142025 dataset includes sample annotations for early and advanced DN provided by the original authors, based on clinical criteria including estimated glomerular filtration rate (eGFR) and urinary albumin-to-creatinine ratio (UACR). All data processing and analyses were performed using R software (version 4.3.3). Raw expression data (CEL files) were background corrected and normalized using the robust multiarray average (RMA) method implemented in the affy package. Probe-to-gene symbol mapping was performed using corresponding platform annotation files: GPL571 for GSE30528 and GSE30529 and GPL17586 for GSE96804. For datasets that did not provide raw data, preprocessed expression matrices were used directly after normalization was verified.

Differential expression analysis between the disease and control groups was conducted using the limma package, with a linear model incorporating empirical Bayes moderation. Batch effects were adjusted using the removeBatchEffect function when necessary. The threshold for significance was set at a |log2-fold change| ≥ 1 and an adjusted P value (padj) < 0.01. Volcano plots were generated using ggplot2.

Functional enrichment analyses, including Gene Ontology (GO) biological process analysis and Kyoto Encyclopedia of Genes and Genomes (KEGG) pathway analysis, were performed using the clusterProfiler package. Gene set enrichment analysis (GSEA) was also conducted using clusterProfiler with default parameters, and FDR < 0.05 was the threshold for significantly enriched gene sets.

Weighted gene coexpression network analysis (WGCNA) was performed using the WGCNA package. Briefly, genes whose median absolute deviation (MAD) was in the top 50% were retained. A signed adjacency matrix was constructed using a soft-thresholding power (β) selected on the basis of a scale-free topology fit (R² > 0.8). The adjacency matrix was transformed into a topological overlap matrix (TOM), and hierarchical clustering was used to identify modules (minimum module size = 30). Module-trait relationships were assessed by correlating module eigengenes with disease status (control, early DN, and advanced DN for kidney data; control, diabetes without DR, and DR for PBMC data). Modules with the highest correlation were selected for further analysis.

Protein-protein interaction (PPI) networks were constructed using the Search Tool for the Retrieval of Interacting Genes/Proteins (STRING) database version 12.0, with a confidence score threshold > 0.7. Networks were visualized and analyzed using Cytoscape software (version 3.9.1). Hub genes within each module were identified using the CytoHubba plugin with the maximal clique centrality (MCC) algorithm.

### Single-Cell RNA Sequencing Data Acquisition and Processing

#### DN scRNA-seq Data (GSE216510)

Mouse kidney scRNA-seq data from diabetic nephropathy models and healthy controls were obtained from GSE216510. Quality control was performed by the original authors. The Seurat package (version 4.3.0) was used for downstream analysis. Data were normalized using the NormalizeData function with default parameters. The top 2000 highly variable genes were identified using FindVariableFeatures. The data were scaled using ScaleData, and principal component analysis (PCA) was performed. The first **17** principal components were used for uniform manifold approximation and projection (UMAP) dimensionality reduction and graph-based clustering (Louvain algorithm) with a resolution parameter of **0.2**. Cell types were manually annotated on the basis of canonical marker genes (e.g., *Pecam1* for endothelial cells, *Pdgfrb* for fibroblasts/pericytes, *Epcam* for epithelial cells, *Ptprc* for immune cells).

#### DR scRNA-seq Data (GSE178121)

Mouse DR scRNA-seq data were obtained from GSE178121. Quality control was performed by filtering out cells with < 200 or > 9000 detected genes or with a mitochondrial gene percentage > 15%. The data were normalized, variable features (2000) were identified, and scaling was performed as described above. PCA was conducted, and the first 20 principal components were used for UMAP and clustering (resolution = 0.01). Cell types were manually annotated using murine markers. Batch correction across samples was performed using the Harmony package.

For both datasets, differential expression between cell types or conditions was performed using the FindAllMarkers or FindMarkers functions with the following thresholds: log2FC > 0.25, p value < 0.05, and minimum expression fraction > 0.1. Visualization was performed using VlnPlot, FeaturePlot, and DoHeatmap.

### Protein-Protein Interaction (PPI) Network Analysis

As described in Section 2.1, PPI networks were constructed using STRINGdb v12.0 (confidence > 0.7). The CytoHubba plugin (MCC algorithm) in Cytoscape 3.9.1 was used to identify hub proteins within each module.

### Machine Learning Framework for Predictive Model Construction

To develop robust and generalizable clinical prediction models, we used an ensemble machine learning approach. For both DN and DR, we derived feature genes as the intersection of disease-associated WGCNA module genes and differentially expressed genes (DEGs). The expression matrix of these feature genes was used as input.

We systematically constructed 120 combinatorial machine learning models using the caret and mlr3 packages in R, as well as custom Python scripts (Python 3.10). The base learners included regularized logistic regression (elastic net), support vector machine (SVM) with radial kernel, k-nearest neighbors (kNN), linear discriminant analysis (LDA), random forest, gradient boosting machine (GBM), extreme gradient boosting (XGBoost), and naive Bayes.

**Training and validation scheme:** 1) The training sets were GSE142025 for DN and GSE221521 for DR (split 80:20 for internal validation). 2) GSE96804 served as the independent external validation set for DN, whereas the remaining 20% of GSE221521 served as the internal hold-out test set for DR.

**Cross-validation:** Five-fold cross-validation (repeated 3 times) was performed within the training set. ROC AUC was used as the primary optimization metric. For each base model, out-of-fold (OOF) predictions were generated.

**Model combination and ensemble learning:**We ranked the base models by their OOF AUC and selected the top N models (N = 3, 4, 5) as candidate base models. For each combination of k models (k = 1, 2, 3), we constructed ensembles using two fusion strategies: soft voting (simple average) and the average of the predicted probabilities from the selected models.

AUC-weighted average was defined as the weighted average where the weight of each model was proportional to its OOF AUC. Weights were normalized to sum to 1.

For each ensemble, we computed the 1) training OOF AUC, i.e., the ROC AUC based on combined OOF probabilities and training labels, and 2) validation AUC, i.e., the ROC AUC based on predictions on the independent external validation set for DN or the internal hold-out test set for DR.

The final model performance was ranked by the corresponding validation AUC (external validation for DN and internal hold-out test set validation for DR). The OOF AUC was used to monitor overfitting.

### Cell-Communication Analysis

Cell-communication analysis was performed using the CellChat package in R. For each dataset (DN and DR), we created a CellChat object using the createCellChat function, providing the normalized expression matrix and cell type annotations. A mouse ligand-receptor database (CellChatDB.mouse) was used for both DN and DR data. The data were preprocessed using subsetData, and overexpressed genes and interactions were identified with identifyOverExpressedGenes and identifyOverExpressedInteractions. The communication probabilities were computed using computeCommunProb (triMean method, population.size = TRUE). Pathways with significant communication were inferred using computeCommunProbPathway. Aggregated networks were obtained using aggregateNet. To compare the communication patterns between the disease and control groups, we performed differential interaction analysis using the netAnalysis_computeCentrality and rankNet functions. Visualization included circle plots, heatmaps, and chord diagrams.

### Pseudotime Trajectory Analysis

Pseudotime analysis was performed using the Monocle2 package. For DN, **fibroblasts and endothelial cells** were analyzed; for DR, **endothelial cells and pericytes/SMCs** were analyzed. We extracted the raw count matrix from the Seurat object and constructed a CellDataSet object using the newCellDataSet function with expressionFamily = negbinomial.size(). Genes expressed in at least 3 cells were retained. Trajectory-building genes were selected as those that were significantly differentially expressed across clusters (Wilcoxon test, q < 0.1). Dimensionality reduction was performed using the DDRTree method (max_components = 3, num_dim = 20). Cells were ordered in pseudotime, and branch points were identified. The expression dynamics of *COL1A2* and *APOLD1* along pseudotime were visualized using plot_genes_in_pseudotime.

### *In silico* Knockout Analysis Using scTenifoldKnk

To investigate the potential regulatory role of COL1A2 in disease-relevant cell populations, we performed an *in silico* gene knockout analysis using the scTenifoldKnk framework. Fibroblasts from the DN scRNA-seq dataset and vascular smooth muscle cells/pericytes from the DR scRNA-seq dataset were extracted for cell type-specific perturbation analysis. The raw single-cell expression matrix of each selected cell population was used as input, and Col1a2 was specified as the target gene for virtual knockout.

The scTenifoldKnk pipeline reconstructs single-cell gene regulatory networks under the wild-type and target-gene knockout states and then compares the two network structures through tensor decomposition and manifold alignment. This approach identifies genes whose regulatory network positions are most strongly perturbed after COL1A2 knockout, rather than relying solely on conventional differential expression analysis. For each cell population, the wild-type regulatory network was first inferred from the original expression matrix. A COL1A2-knockout network was then generated *in silico*, and the manifold distance between the wild-type and knockout networks was calculated for each gene. Genes showing significant network perturbation after COL1A2 knockout were ranked according to their perturbation scores and adjusted statistical significance.

The top perturbed genes identified in DN fibroblasts and DR vascular smooth muscle cells/pericytes were visualized and interpreted as putative downstream response genes of COL1A2. This analysis was used to explore the context-dependent regulatory consequences of COL1A2 dysregulation in diabetic nephropathy and diabetic retinopathy.

### Validation by Western Blotting

**Animals:** Type 2 diabetes was modeled using db/db mice (BKS.Cg-Dock7m+/+ Leprdb/J). Age-matched db/m+ mice served as controls. The mice were housed under specific pathogen-free (SPF) conditions with *ad libitum* access to sterilized feed and water. Diabetes was confirmed by a blood glucose concentration > 16.7 mmol/L. The mice were euthanized at 1, 3, and 6 months post-diabetes confirmation. Retinas and kidney cortices were rapidly dissected, snap-frozen in liquid nitrogen, and stored at -80 °C until use. Three mice per group were used for each time point, and all Western blot experiments were repeated independently three times. Representative blots from one independent experiment are shown. All procedures adhered to the ARVO Statement for the Use of Animals in Ophthalmic and Vision Research and were approved by the Animal Ethics Committee of the Second Affiliated Hospital, Zhejiang University School of Medicine (Approval ID: 2026002).

### Protein extraction and Western blotting

Tissues were homogenized in RIPA buffer (Beyotime, China) supplemented with protease inhibitor cocktail (Roche, Switzerland). The protein concentration was determined using a BCA assay (Thermo Fisher Scientific, USA). Equal amounts of protein (30 μg) were separated by 10% SDS-PAGE and transferred to PVDF membranes (Millipore, USA). The membranes were blocked with 5% nonfat milk in TBST for 1 h at room temperature and then incubated overnight at 4 °C with the following primary antibodies: anti-COL1A2 (Abcam, ab96723, 1:1000), anti-APOLD1 (Santa Cruz, sc-515656, 1:500), and anti-β-actin (Cell Signaling Technology, #4970, 1:5000) as a loading control. After being washed, the membranes were incubated with HRP-conjugated secondary antibodies (1:5000; Jackson ImmunoResearch, USA) for 1 h at room temperature. Signals were visualized using enhanced chemiluminescence (ECL) reagent (Millipore, USA) and imaged with a ChemiDoc MP system (Bio-Rad, USA). Band intensities for target proteins were quantified using ImageJ software (NIH, USA) and normalized to the band intensity for β-actin.

### Statistical Analysis

All data processing, statistical analyses, and visualization were performed using R 4.3.3, Python 3.10, and CentOS. For the Western blot data, the results are expressed as the mean ± SEM. Comparisons among multiple time points were conducted using one-way ANOVA followed by Tukey's post hoc test. Comparisons between two groups were performed using two-tailed Student's t tests. For cell proportion analysis, the chi-square test was applied. A p value < 0.05 was considered to indicate statistical significance.

## Results

### Bulk Transcriptome Analysis of DN and DR Revealed Disease-Specific Molecular Signatures

**Figure 1:**
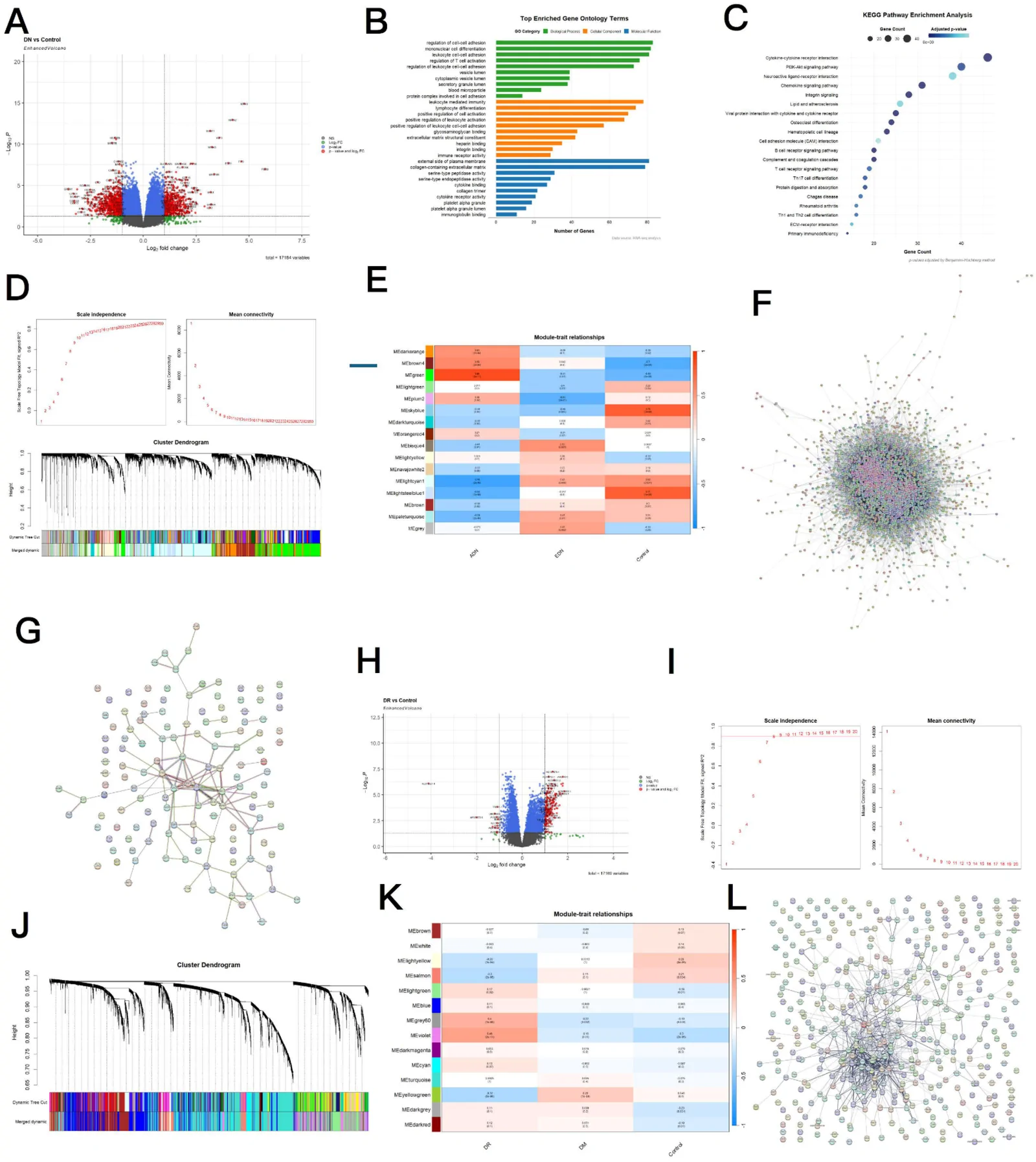
** Bulk transcriptome analysis of DN and DR. (A)** Volcano plot of DEGs in DN vs. control kidney tissue. **(B)** GO enrichment of DEGs in DN. **(C)** KEGG enrichment of DEGs in DN. **(D)** WGCNA soft-thresholding power selection in DN dataset. **(E)** Module-trait heatmap showing correlation between modules and disease states (control, EDN, ADN). **(F)** PPI network of MEgreen module (associated with ADN). **(G)** PPI network of MEbisque4 module (associated with EDN). **(H)** Volcano plot of DEGs in DR vs. control PBMC samples. **(I)** WGCNA soft-thresholding power selection in DR dataset. **(J)** Module-trait relationships showing MEviolet correlated with DR and MEyellowgreen with DM. **(K)** Module-trait heatmap for DR dataset. **(L)** PPI network of MEviolet module.

We first performed differential expression analysis using kidney tissue samples from DN samples versus control samples (GSE142025). A total of 506 genes were upregulated and 660 were downregulated in DN samples compared with control samples (|log2FC| ≥ 1, padj < 0.01; Figure 1A). GO enrichment analysis of the DEGs revealed significant enrichment in biological processes related to cell-cell adhesion, mononuclear cell adhesion, leukocyte cell adhesion, and regulation of T cell activation (Figure 1B). The results of the KEGG pathway enrichment analysis indicated significant enrichment in cytokine-cytokine receptor interactions, the PI3K-Akt scRNA-seq signaling pathway, neuroactive ligand-receptor interactions, and chemokine signaling pathways (Figure 1C). These findings indicate that immune-inflammatory processes play a prominent role in DN.

Given that the dataset included three groups (control, early DN (EDN), and advanced DN (ADN)), we performed WGCNA to identify gene modules associated with different disease states. The soft-thresholding power was set to 6 on the basis of the scale-free topology fit (R² = 0.9) (Figure 1D). Module-trait correlation analysis revealed that the MEgreen module was most strongly associated with ADN and that the MEbisque4 module was most strongly associated with EDN (Figure 1E). PPI network analysis of the MEgreen and MEbisque4 modules revealed hub genes (Figure 1F and 1G). The MEgreen module contained genes enriched in ECM-receptor interactions and focal adhesion, which is consistent with advanced fibrotic changes, whereas the MEbisque4 module contained genes enriched in metabolic processes, suggesting early metabolic dysregulation.

Similarly, we analyzed PBMCs from DR samples and control samples (GSE160306). Differential expression revealed 166 upregulated and 13 downregulated genes in DR (Figure 1H). WGCNA was performed using this dataset (soft-thresholding power = 5; Figure 1I). Module-trait analysis revealed that the MEviolet module was most strongly correlated with DR and that the MEyellowgreen module was most strongly correlated with diabetes without DR (Figure 1J-K). The PPI network for the MEviolet module revealed hub genes (Figure 1L). The MEviolet module contained genes enriched in the immune response and cytokine signaling, suggesting the presence of systemic immune alterations in DR.

### Machine Learning Identified Robust Predictive Signatures for DN and DR

**Figure 2:**
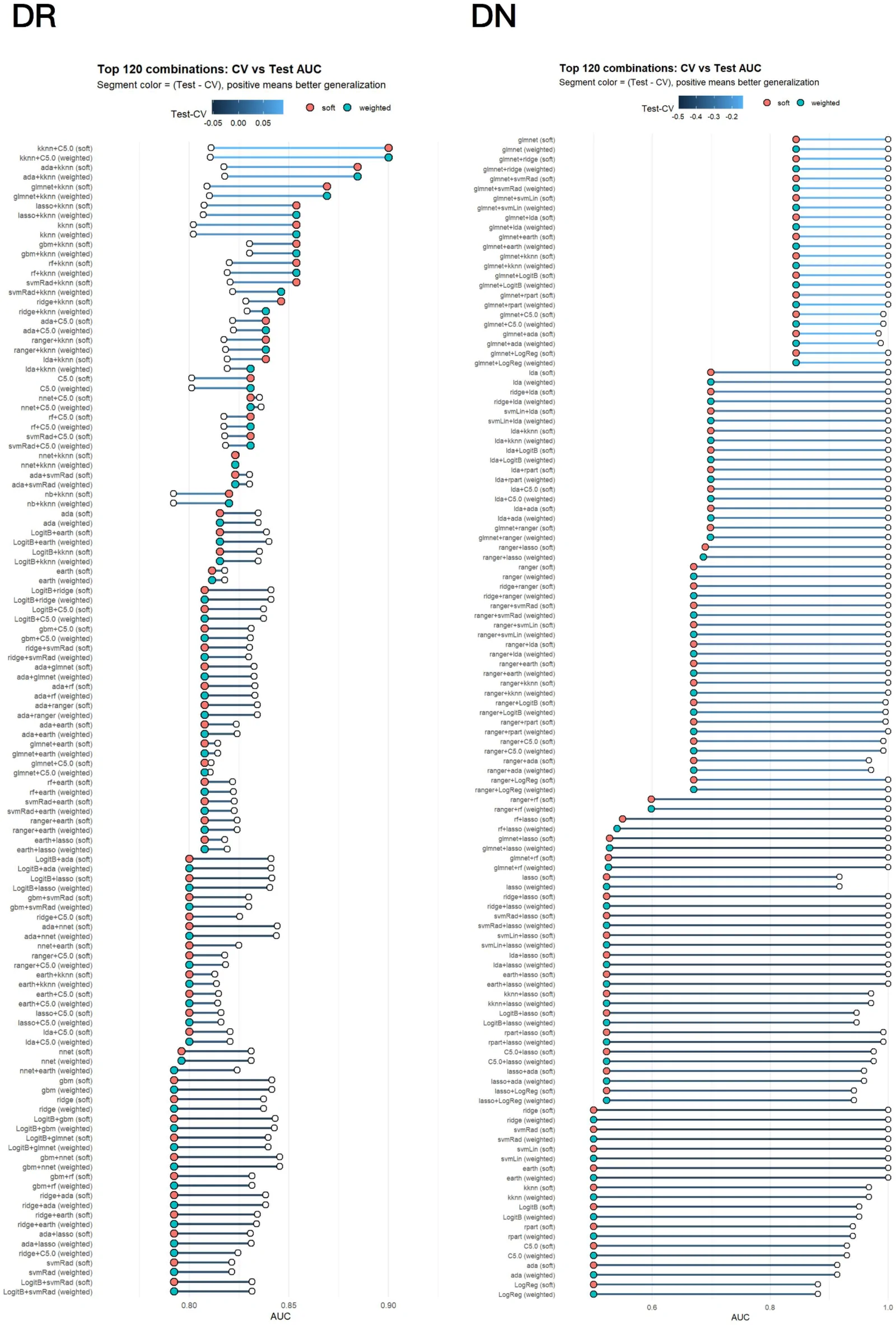
** Machine learning predictive models. (A)** Comparison of the discriminative performance of candidate DR model configurations in the training and internal hold-out validation sets. The plot displays the 120 model-voting specifications with the highest AUCs in the internal hold-out validation set. For each specification, the open circle on the left represents the OOF AUC obtained by repeated cross-validation in the training set, whereas the colored circle on the right represents the AUC observed in the internal hold-out validation set. Red and turquoise circles denote equal-weight soft voting and voting weighted by the OOF AUCs of the constituent learners, respectively. The connecting segment and its color represent the difference between the internal hold-out and training OOF AUCs (hold-out AUC - OOF AUC). A positive difference indicates only a higher observed AUC in the current held-out sample and should not, by itself, be interpreted as evidence of superior generalizability. This plot provides a descriptive comparison of training and held-out performance; to avoid test-set leakage, hold-out AUCs were not used for final model selection. The final LogitBoost model was prespecified exclusively according to its training OOF AUC. **(B)** Comparison of cross-validation and external test AUCs for candidate single models and two-model combinations. Single models and pairwise combinations were constructed from the 15 base learners with the highest training OOF AUCs. Ensemble probabilities were calculated using either unweighted probability averaging (soft) or probability averaging weighted by the training OOF AUCs of the constituent models (weighted). The figure presents the top 120 model-combination and aggregation-strategy entries ranked in descending order of external test AUC. Each row represents one candidate combination and aggregation strategy. Open circles indicate training OOF cross-validation AUCs, colored filled circles indicate external test AUCs, and horizontal segments connect the two estimates. Segment color represents the generalization gap, calculated as Test AUC minus CV AUC. Values closer to zero indicate greater agreement between training and external performance, whereas negative values indicate lower performance in the external test cohort. Red filled circles denote soft averaging, and cyan filled circles denote weighted averaging. Higher external AUCs together with shorter segments indicate relatively better external generalization. Because entries were ranked using external test AUC, this figure represents an exploratory comparison and was not used for final model selection or model locking.

To develop clinically applicable predictive models, we took the intersection of module genes (from WGCNA) and DEGs as features for machine learning. We systematically constructed 120 combinatorial models as described in the Methods section (Figure 2A-B). For DN combinatorial ML models, the svmRad model was locked using sample-level outer-OOF predictions from the training cohort, without access to external labels or external AUC. The model achieved a training OOF AUC of 1.000 and an OOF Brier score of 0.007. Given the small training cohort of 36 samples, this complete separation indicates strong separability within the available training data but should not be interpreted as an unbiased estimate of performance in unseen populations.

In the independent external cohort of 61 samples, the locked model achieved an AUC of 0.874 (95% CI, 0.786-0.963), indicating good external discrimination (Fig S1A). External probabilities did not collapse: all 61 samples had distinct predicted probabilities, ranging from 0.181 to 0.969. At the prespecified training OOF threshold of 0.434, external sensitivity was 1.000, specificity was 0.050, positive predictive value was 0.683, negative predictive value was 1.000, and accuracy was 0.689. The corresponding confusion matrix comprised 41 true positives, 19 false positives, no false negatives, and one true negative (Fig S1B). Thus, the threshold identified all DN cases but resulted in a high false-positive rate.

Decision curve analysis showed that the model provided greater net benefit than the treat-all and treat-none strategies across an approximate threshold-probability range of 0.19-0.70, suggesting potential clinical utility at intermediate decision thresholds (Fig S1C). A positive net benefit was also retained around the training OOF-locked threshold of 0.434.

Despite its good external discrimination, the model showed suboptimal absolute-risk calibration (Fig S1D). For the raw external probabilities, the Brier score was 0.223 and the five-bin ECE was 0.206. Calibration-in-the-large was -1.414, while the jointly estimated calibration intercept and slope were -6.536 and 3.433, respectively. The external calibration curve indicated systematic overestimation of DN risk. Platt scaling yielded an external Brier score of 0.311 and an ECE of 0.311, while isotonic regression yielded corresponding values of 0.300 and 0.305. Neither method improved upon the raw probabilities.

Overall, the locked model demonstrated good ranking and discrimination in the independent external cohort and showed potential clinical net benefit across a range of decision thresholds. However, the prespecified threshold had low specificity, and the predicted probabilities exhibited substantial calibration drift. The model may therefore be more appropriate as a high-sensitivity screening tool, while its classification threshold and absolute-risk estimates require further validation in larger and more balanced independent cohorts. We also assessed feature importance stability by examining the rank consistency of top predictive genes across cross-validation folds. In both the DN and DR models, COL1A2 and APOLD1 consistently appeared among the top contributors, with limited rank variation across folds, supporting the robustness of these signatures.

For DR combinatorial ML models, to avoid information leakage and optimistic performance estimates arising from validation-guided model selection, the final model was selected exclusively according to out-of-fold predictions generated by repeated five-fold cross-validation, with three repeats, in the training set. The 15 highest-ranked base learners according to their training OOF AUCs were retained, yielding 120 candidate model configurations comprising 15 individual models and 105 pairwise soft-voting combinations. The configuration with the highest training OOF AUC was selected; ties were resolved by prioritizing configurations containing fewer base learners and then by model name. LogitBoost, denoted as LogitB in the analysis code, achieved the highest training OOF AUC of 0.867 and was therefore selected as the final model. Because the selected configuration contained only one base learner, the final model was a single LogitBoost classifier rather than a multi-model ensemble. The internal hold-out validation set was used only for final performance evaluation and was not involved in model selection.

ROC analysis showed that the final LogitBoost model achieved an AUC of 0.777 in the internal hold-out validation set (95% CI, 0.576-0.978; Figure S1D). At the prespecified threshold of 0.719, which was determined exclusively from the training OOF predictions using the Youden index and locked before validation, the model achieved a sensitivity of 0.846 and a specificity of 0.400. Decision curve analysis indicated that the LogitBoost model provided a greater net benefit than the treat-all and treat-none strategies at selected threshold probabilities between approximately 0.34 and 0.64 (Figure S1E). The calibration curves deviated from the ideal line in both the training and internal hold-out validation data, with wide confidence intervals in the held-out set (Figure S1F), indicating that probability calibration remains uncertain and requires confirmation in larger independent external cohorts.

### Cross-Tissue Analysis Identified COL1A2 as a Consistently Upregulated Gene and APOLD1 as a Consistently Downregulated Gene in DN and DR

**Figure 3:**
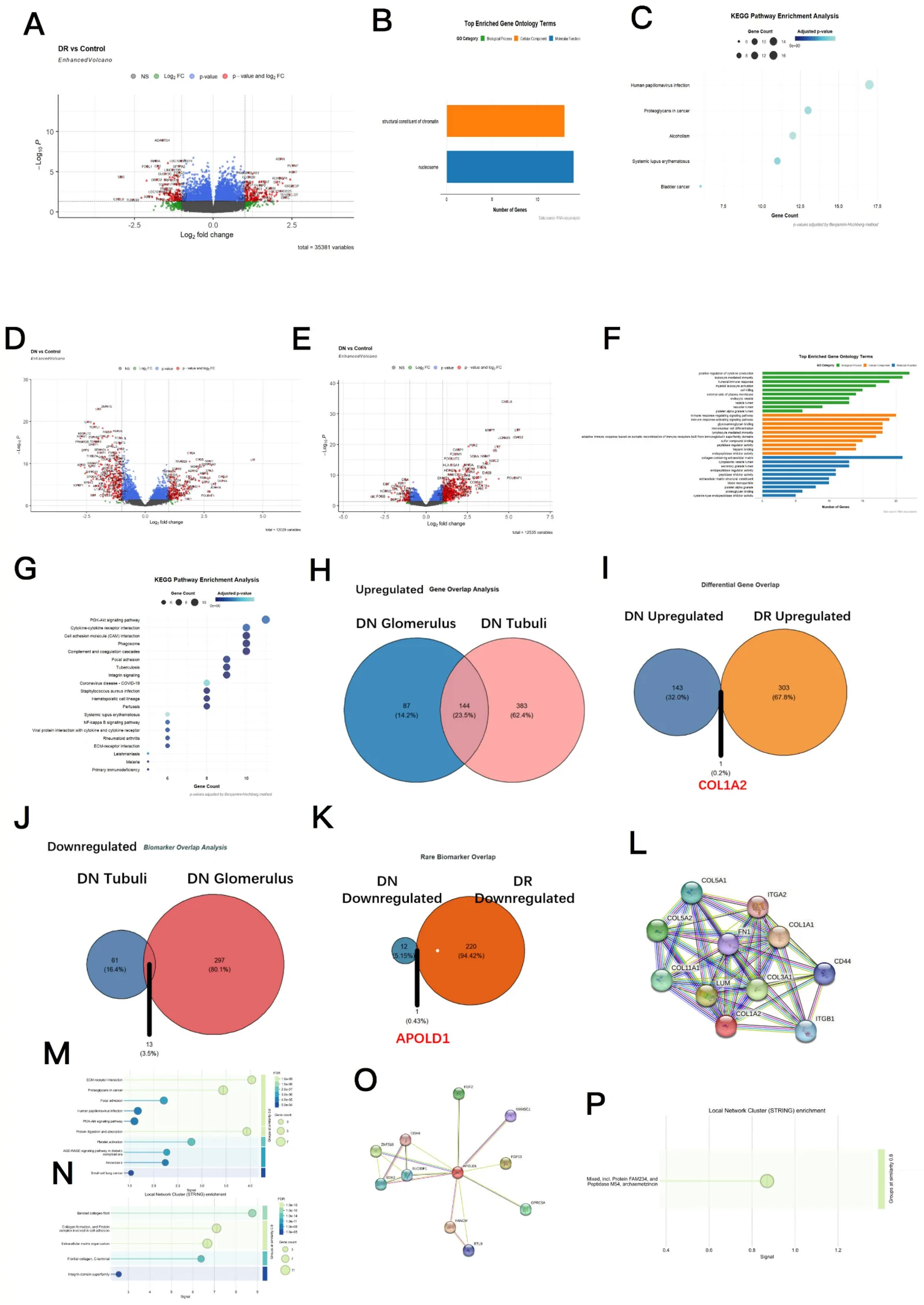
** Cross-tissue comorbidity analysis identifies COL1A2 and APOLD1 as shared genes. (A)** Volcano plot of DEGs in DR vs. control retina. **(B)** GO enrichment of DR DEGs. **(C)** KEGG enrichment of DR DEGs. **(D)** Volcano plot of DEGs in DN glomeruli. **(E)** Volcano plot of DEGs in DN tubules. **(F)** GO enrichment of common DN DEGs (intersection of glomerular and tubular). **(G)** KEGG enrichment of common DN DEGs. **(H)** Venn diagram of upregulated genes in DN (glomerular + tubular) and DR (retina), identifying COL1A2 as the sole common upregulated gene. **(I)** Venn diagram of common upregulated genes. **(J)** Venn diagram of downregulated genes in DN and DR. **(K)** Venn diagram of common downregulated genes, identifying APOLD1. **(L)** PPI network of COL1A2-interacting genes. **(M)** KEGG enrichment of COL1A2-interacting genes. **(N)** STRING enrichment of COL1A2-interacting genes. **(O)** PPI network of APOLD1-interacting genes. **(P)** STRING enrichment of APOLD1-interacting genes.

We next performed differential expression analysis using retinal DR samples and control samples (GSE221521). A total of 304 genes were upregulated, and 221 downregulated (Figure 3A). GO enrichment analysis revealed terms such as structural constituent of chromatin and nucleosome (Figure 3B); KEGG analysis revealed the enrichment of genes in pathways involved in HPV infection, proteoglycans in cancer, and alcoholism (Figure 3C). These terms may reflect epigenetic remodeling and extracellular matrix changes in the diabetic retina.

For DN, we separately analyzed glomerular and tubular compartments (GSE142025). In glomeruli, 315 upregulated and 278 downregulated genes were detected; in tubules, 421 upregulated and 392 downregulated genes were detected (Figures 3D and 3E). The intersection of glomerular and tubular DEGs yielded a set of commonly altered genes. GO analysis revealed the enrichment of these common genes in the positive regulation of cytokine production, leukocyte-mediated immunity, cell killing, and mononuclear cell differentiation (Figure 3F). The enriched KEGG terms included PI3K-Akt, cytokine-cytokine receptor interaction, and cell adhesion molecules (CAMs) (Figure 3G). These pathways underscore the inflammatory and adhesive changes that transcend renal compartments.

To identify shared comorbidity genes, we intersected upregulated genes in DN (glomerular and tubular overlap) and DR (retina). This intersection revealed *COL1A2* as the sole common upregulated gene (Figure 3H-I). The intersection of downregulated genes revealed *APOLD1* as the sole common downregulated gene (Figure 3J-K). The exclusivity of these two genes highlights their potential as core comorbidity drivers.

We then constructed protein-protein interaction (PPI) networks for COL1A2 and APOLD1 using STRINGdb. COL1A2-interacting genes (Figure 3L) were significantly enriched in the ECM-receptor interaction, focal adhesion, and PI3K-Akt pathways (Figure 3M-N). APOLD1-interacting genes, including the protein FAM234 and peptidase M54 (archaemetzincin) (Figure 3O), were enriched in mixed functions (Figure 3P). The APOLD1 network may play a role in protein degradation and vascular remodeling.

### Single-Cell RNA Sequencing Reveals the Cell Type-Specific Localization of COL1A2 and APOLD1 in Mouse Models

**Figure 4:**
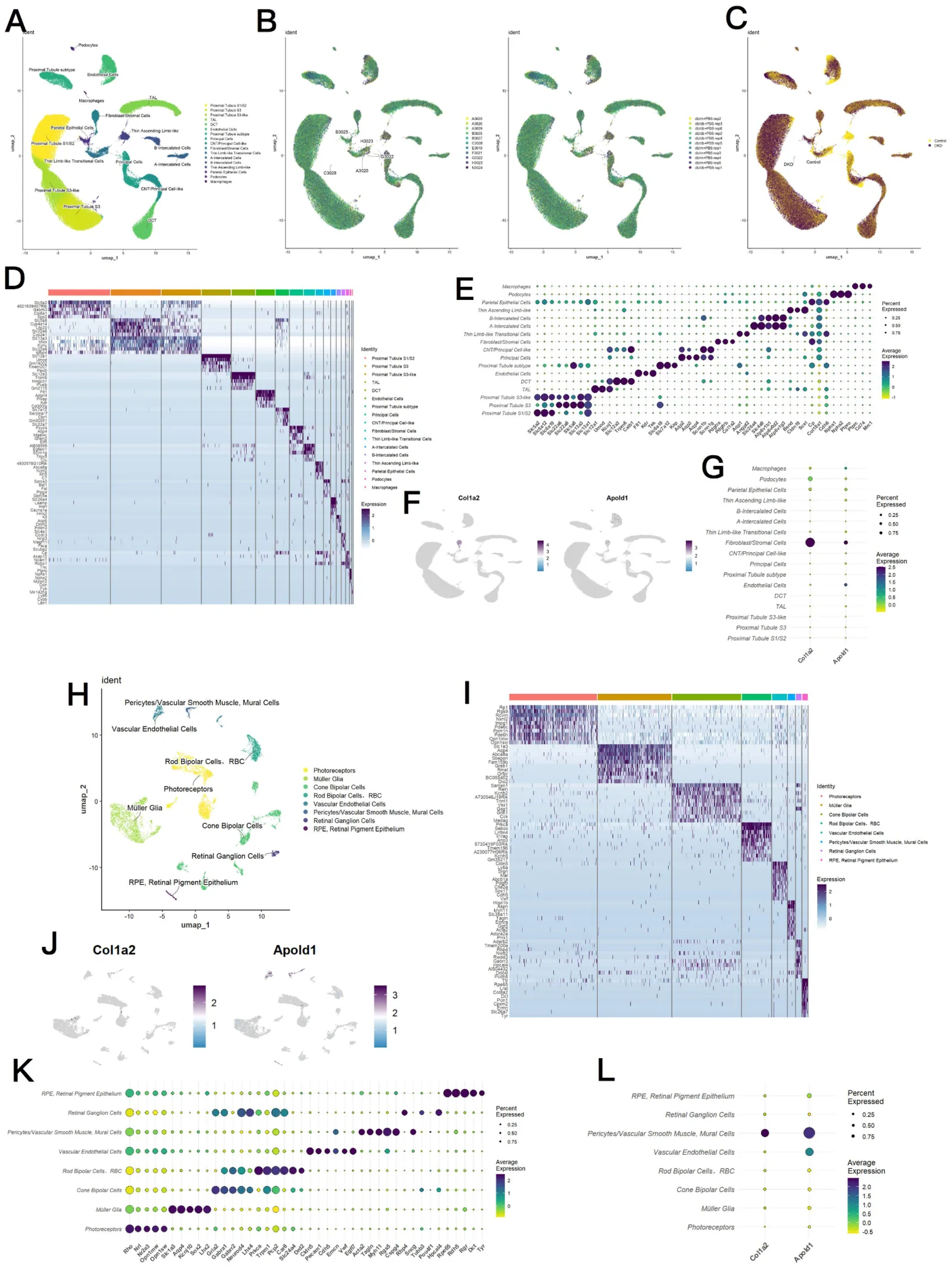
** Single-cell RNA sequencing reveals cell type-specific expression of COL1A2 and APOLD1 in mouse models. (A)** UMAP plot of cell types in DN mouse kidney (GSE216510). **(B)** UMAP colored by sample origin. **(C)** UMAP colored by group (DN vs. control). **(D)** Heatmap of canonical marker genes for each cell type in DN dataset. **(E)** Dot plot of marker genes for cell type annotation in DN. **(F)** Feature plots showing COL1A2 and APOLD1 expression in DN kidney — specific to fibroblasts. **(G)** Dot plot of COL1A2 and APOLD1 expression across cell types in DN. **(H)** UMAP plot of cell types in DR mouse retina (GSE178121). **(I)** Heatmap of marker genes in DR dataset. **(J)** Feature plots showing COL1A2 and APOLD1 expression in DR retina — predominantly in pericytes/SMCs. **(K)** Dot plot of marker genes for cell type annotation in DR. **(L)** Dot plot of COL1A2 and APOLD1 expression across cell types in DR.

To further investigate the cellular context of these two genes, we analyzed scRNA-seq data from mouse DN kidney (GSE216510) and mouse DR retina (GSE178121). To further investigate the cellular context of these two genes, we analyzed scRNA-seq data from mouse DN kidney (GSE216510) and mouse DR retina (GSE178121). GSE216510 contained 23,796 cells before quality control and 21,543 cells after filtering; GSE178121 contained 19,562 cells before quality control and 10,410 cells after filtering. After quality control and clustering, we annotated cell types on the basis of canonical markers (Figures 4A--E for DN; Figures 4H--I, K for DR). In DN kidney samples, both *COL1A2* and *APOLD1* were specifically expressed in fibroblasts (Figures 4F-G). In DR retina samples, COL1A2 and APOLD1 were predominantly expressed in pericytes/vascular smooth muscle cells, and *APOLD1* was also expressed in endothelial cells (Figures 4J, L). This differential cellular mapping — fibroblasts in the kidney versus pericytes/endothelial cells in the retina — suggests that although both complications involve vascular pathology, the primary affected cell types differ, possibly reflecting the distinct cellular composition of the two organs.

### Cell Proportion and Pseudotime Trajectory Analyses Revealed Dynamic Changes in Key Cell Populations and Gene Expression

**Figure 5:**
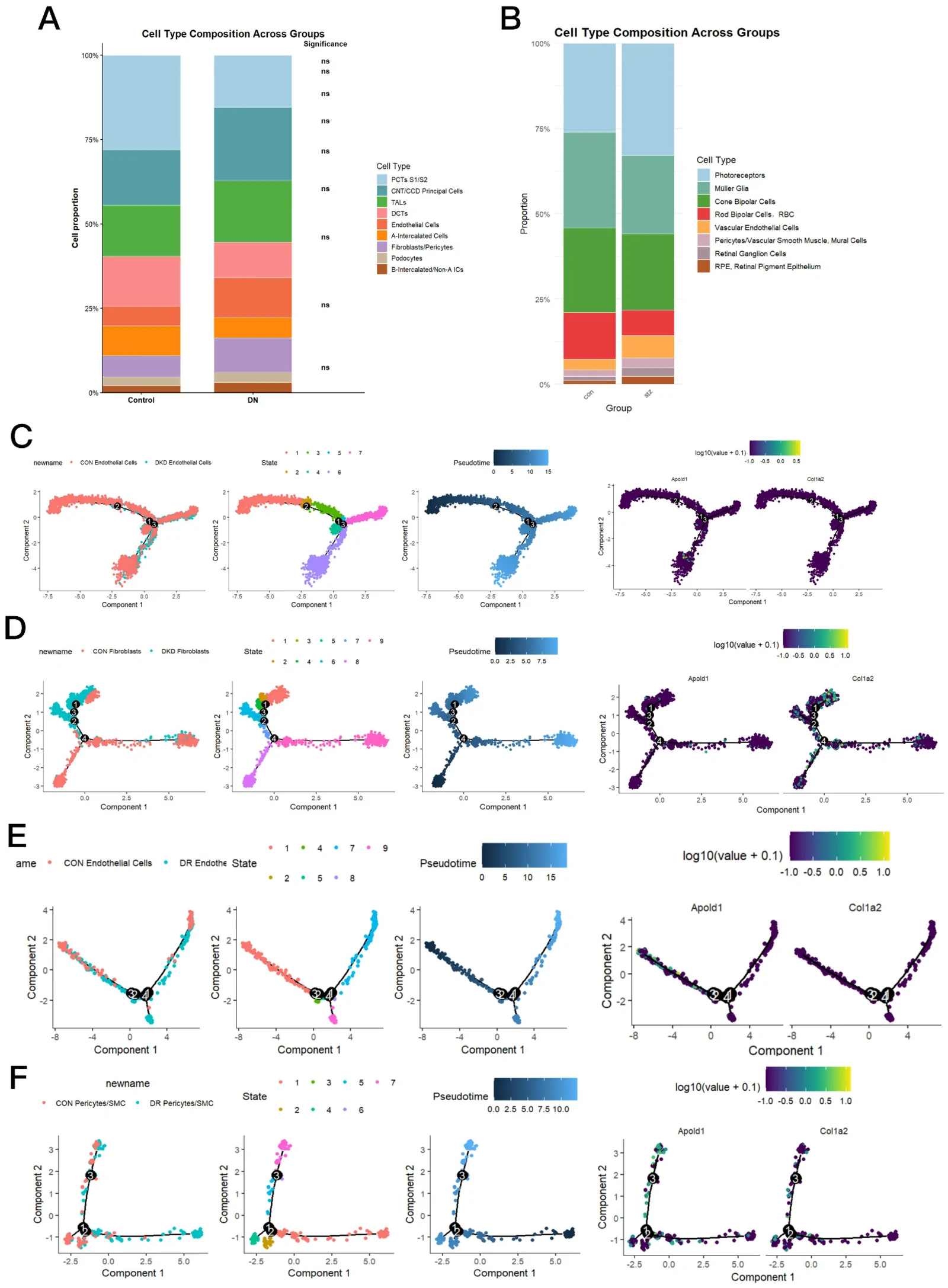
** Cell proportion, pseudotime trajectory (A)** Stacked bar plot of cell type proportions in DN vs. control. Statistical significance was assessed using chi-square test; P < 0.05 for the comparisons described in the main text. **(B)** Stacked bar plot of cell type proportions in DR vs. control. Because each group contained only one animal sample, statistical comparisons were not performed. **(C)** Pseudotime trajectory of endothelial cells in DN and Expression dynamics of APOLD1 and COL1A2 along pseudotime (integrated). **(D)** Pseudotime trajectory of fibroblasts in DN and Expression dynamics of APOLD1 and COL1A2 along pseudotime (integrated). **(E)** Pseudotime trajectory of endothelial cells in DR and Expression dynamics of APOLD1 and COL1A2 along pseudotime (integrated). **(F)** Pseudotime trajectory of pericytes/SMCs in DR and Expression dynamics of APOLD1 and COL1A2 along pseudotime (integrated).

We compared cell proportions between the disease and control groups. In DN, the proportions of fibroblasts and endothelial cells significantly increased, whereas those of proximal tubule S1/S2 and S3 cells decreased (Figure 5A). In DR, photoreceptors and vascular endothelial cells increased, whereas RBCs decreased (Figure 5B), though statistical testing was not performed for DR cell proportion differences because each group contained only one biological sample in the GSE178121 dataset.

To understand the temporal dynamics of *COL1A2* and *APOLD1*, we performed pseudotime trajectory analysis on DN endothelial cells, DN fibroblasts, DR endothelial cells, and DR pericytes/SMCs. In DN endothelial cells, pseudotime ordering revealed three major states (Figure 5C). In DN fibroblasts, a distinct trajectory was observed (Figure 5D). In DR endothelial cells (Figure 5E) and DR pericytes/SMCs (Figure 5F), additional trajectories were identified. The expression dynamics of *APOLD1* and *COL1A2* along pseudotime are summarized in Figure 5G. *APOLD1* was highly expressed in early pseudotime in most cell types, whereas *COL1A2* expression peaked in late pseudotime, particularly in DN fibroblasts and DR pericytes/SMCs. The early downregulation of APOLD1 at 1 month post-diabetes onset is of particular clinical interest, as it precedes visible pathological changes and suggests that APOLD1 may serve as a molecular indicator of subclinical DR or a pre-DR state.

**Figure 6:**
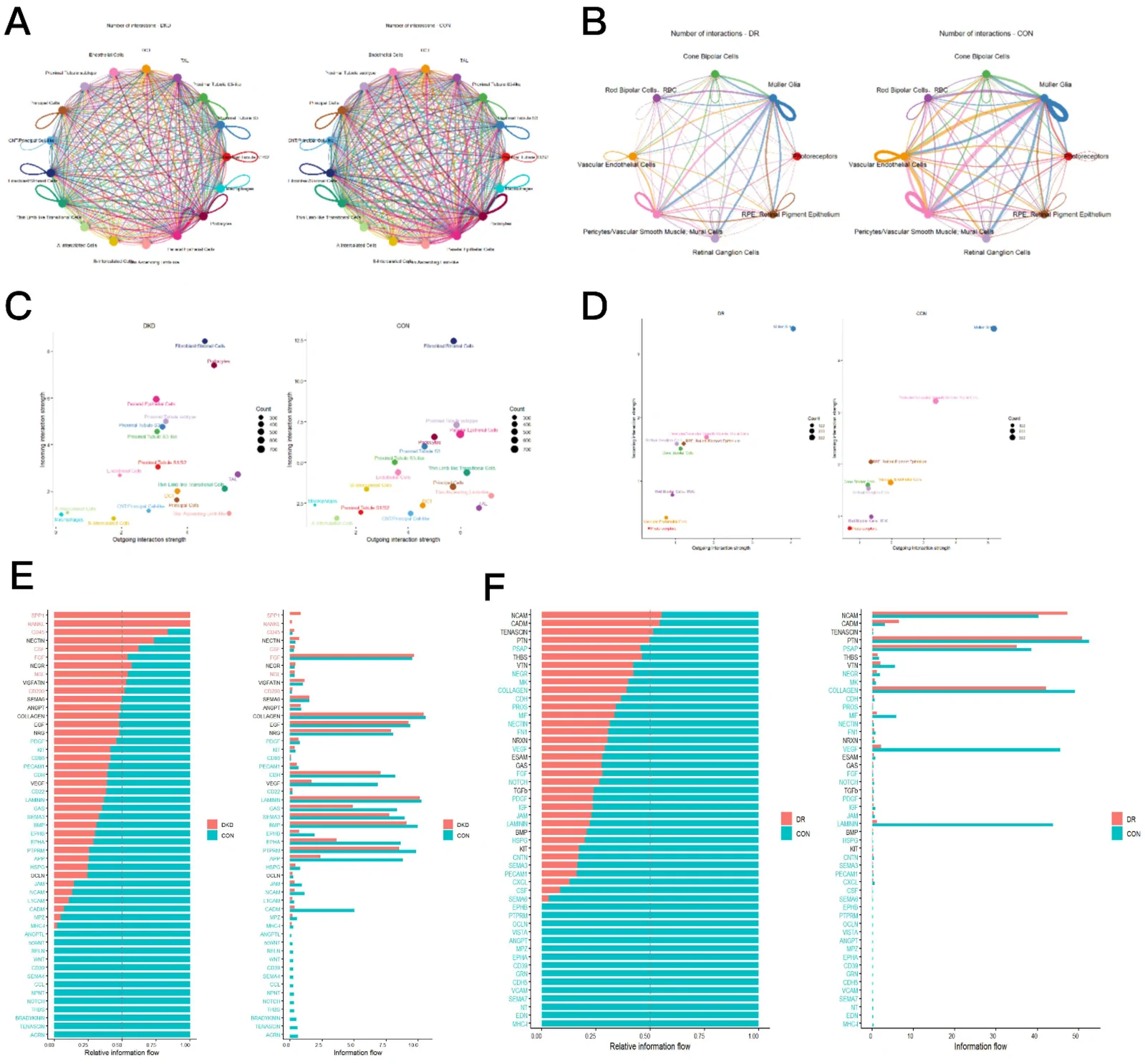
** Cell Communications. (A)** Cell-communication circle plot in DN vs. control. **(B)** Cell-communication circle plot in DR vs. control. **(C)** Coordinate plot of cell-communication in DN. **(D)** Coordinate plot in DR. **(E)** Heatmap of differential pathway signaling intensity in DN vs. control. **(F)** Heatmap of differential pathway signaling intensity in DR vs. control.

Cell-communication analysis revealed extensive network dysregulation. In DN, compared with controls, SPP1, RANKL, CD45, CSF, and FGF pathways were significantly activated, whereas WNT, ARGN, and NOTCH pathways were suppressed (Figures 6A, 6B, 6C). In DR, MHC-I, EDN, LAMININ, and VEGF pathways were significantly suppressed (Figures 6D, 6E, 6F).

### APOLD1 and COL1A2 lack a direct coordinated expression relationship at the single-cell level but are assigned to distinct co-expression modules in DR-associated vascular mural cells

To further determine whether the APOLD1-associated early axis and the COL1A2-associated late axis are directly linked at the molecular level, we first examined the single-cell expression correlation between APOLD1 and COL1A2 in DN Fibroblasts/Pericytes. As shown in Figure S2A, essentially no correlation was detected in the control group (Spearman ρ = -0.022, P = 0.529, n = 845), and no significant correlation was observed in the DN group (Spearman ρ = 0.027, P = 0.453, n = 803). These findings indicate that APOLD1 and COL1A2 do not exhibit a stable coordinated expression pattern in DN-associated Fibroblasts/Pericytes.

We next applied hdWGCNA to determine whether the two genes participate in the same or distinct co-expression programs. Based on the scale-free topology fit and network connectivity characteristics, a soft-thresholding power of 8 was selected for network construction in DN Fibroblasts/Pericytes (Figure S2B), and multiple co-expression modules were subsequently identified (Figure S2C). However, both APOLD1 and COL1A2 were assigned to the unclassified grey category rather than to robust non-grey modules. Thus, the DN Fibroblasts/Pericytes network provided no evidence that APOLD1 and COL1A2 constitute a stable shared co-expression module or a coordinated transcriptional program.

We further examined their relationship in DR-associated Pericytes/Vascular Smooth Muscle/Mural Cells. As shown in Figure S2D, no significant correlation between Apold1 and Col1a2 was observed in either the control group (Spearman ρ = 0.104, P = 0.342, n = 85) or the DR group (Spearman ρ = 0.055, P = 0.458, n = 181). Consistent with the DN findings, the single-gene expression data therefore did not support a stable direct coordinated relationship between Apold1 and Col1a2.

Subsequent hdWGCNA analysis selected a soft-thresholding power of 6 according to the scale-free topology characteristics (Figure S2E) and identified multiple co-expression modules in DR-associated Pericytes/Vascular Smooth Muscle/Mural Cells (Figure S2F). Notably, Apold1 was assigned to the yellow module, whereas Col1a2 was assigned to the turquoise module, indicating that the two genes participate in distinct co-expression programs rather than a shared stable module.

Further analysis of the two target modules showed that the Apold1-associated yellow module and the Col1a2-associated turquoise module exhibited a moderate positive correlation at the module eigengene level (Spearman ρ = 0.402, P < 0.001; Figure S2G). Therefore, although Apold1 and Col1a2 showed no significant direct correlation at the single-gene level and were assigned to distinct co-expression modules, their broader transcriptional programs nevertheless displayed a degree of coordinated variation.

Collectively, these findings do not support a simple direct linear regulatory relationship or stable synchronous co-expression between APOLD1/Apold1 and COL1A2/Col1a2. Instead, particularly in DR-associated vascular mural cells, the two genes were assigned to distinct co-expression modules, suggesting that they represent molecularly distinct but not completely independent transcriptional programs. Together with the stage-specific expression dynamics observed in the pseudotime analyses, these findings are more consistent with an APOLD1-associated early vascular-response axis and a COL1A2-associated late stromal/extracellular-matrix remodeling axis than with a directly demonstrated molecular switch.

### *In silico* COL1A2 Knockout Revealed Context-Dependent Transcriptional Responses

**Figure 7:**
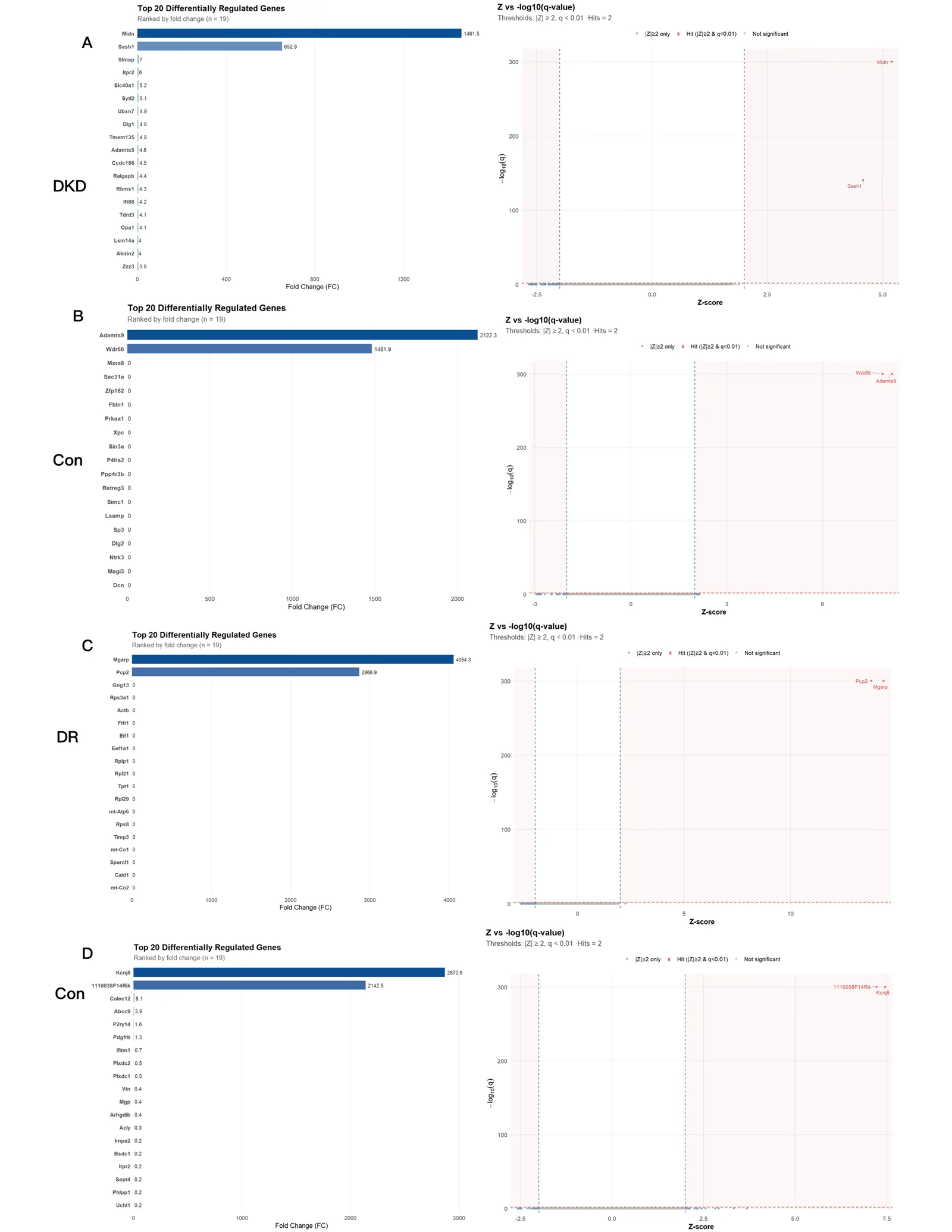
***In silico* knockout of COL1A2 in fibroblasts and SMCs.** Top 20 upregulated genes after virtual COL1A2 knockout in: **(A)** DN fibroblasts (disease group). **(B)** Control fibroblasts. **(C)** DR SMCs (disease group). **(D)** Control SMCs.

To explore the potential downstream effects of COL1A2 dysregulation, we performed *in silico* COL1A2 knockout in DN fibroblasts and DR SMCs. In DN fibroblasts, the genes whose expression was most upregulated after COL1A2 knockout included immune-related markers such as *Midn* and *Sash1* (Figures 7A-B), suggesting that COL1A2 perturbation may be linked to immune infiltration or inflammatory programs in the DN microenvironment. In contrast, in DR SMCs, the top responsive genes included *Mgarp* and *Pcp2* (Figures 7C-D), indicating a vascular/stress-related transcriptional response. These results provide candidates for subsequent experimental validation focusing on ECM-receptor interactions, adhesion, and inflammatory pathways.

### Cell-Communication Analysis Revealed Divergent Signaling Remodeling in DN and DR

**Figure 8:**
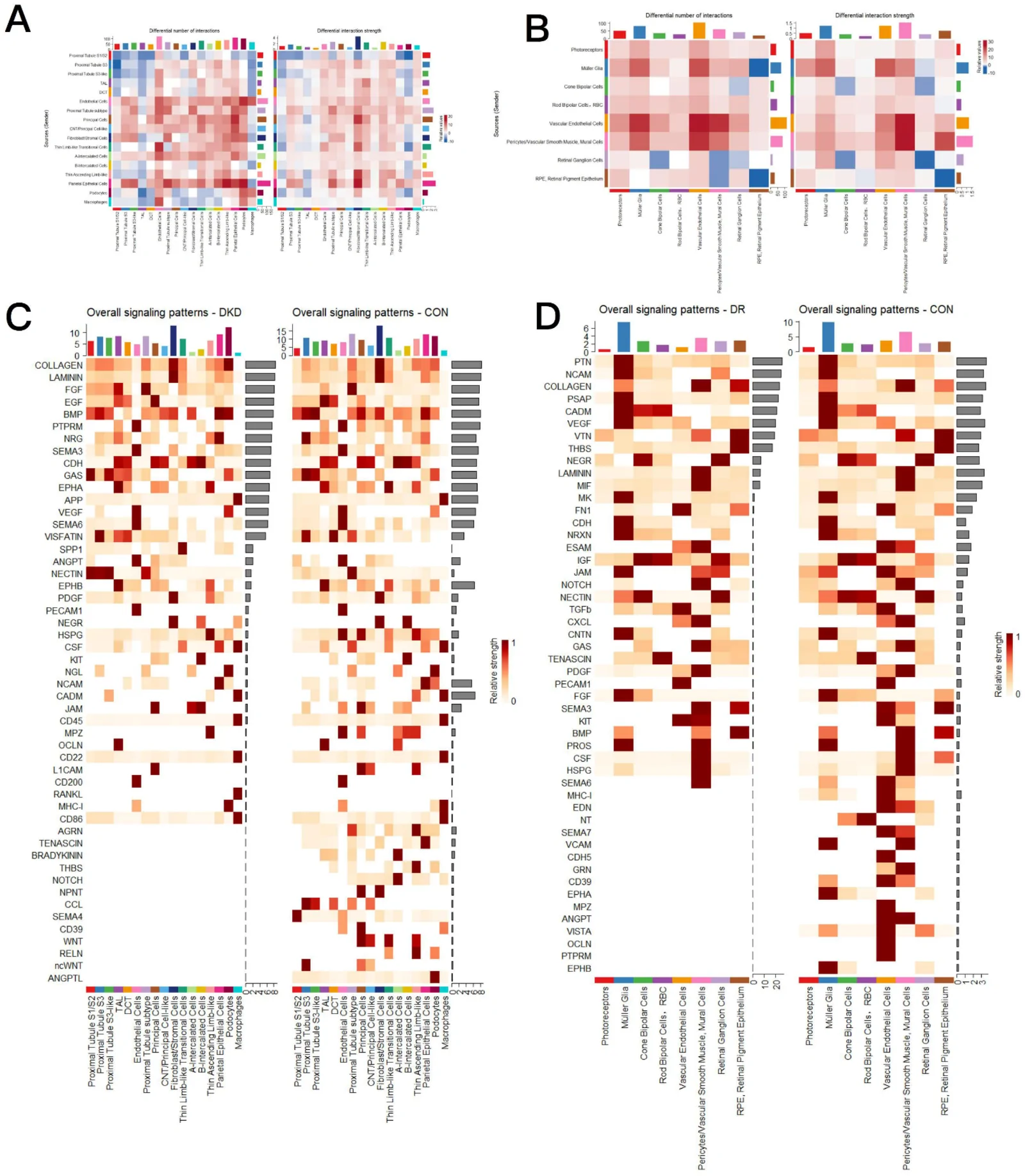
** Cell-communication analysis (detailed for DR). (A)** Heatmap of communication strength in DN vs. control. **(B)** Heatmap of communication strength in DR vs. control. **(C)** Heatmap of pathway-level communication strength in DN vs. control. **(D)** Heatmap of pathway-level communication strength in DR vs. control. **(E)** Dot plot of downregulated ligand-receptor pairs in DR (receivers: SMCs/endothelial cells). **(F)** Dot plot of upregulated ligand-receptor pairs in DR.

**Figure 9:**
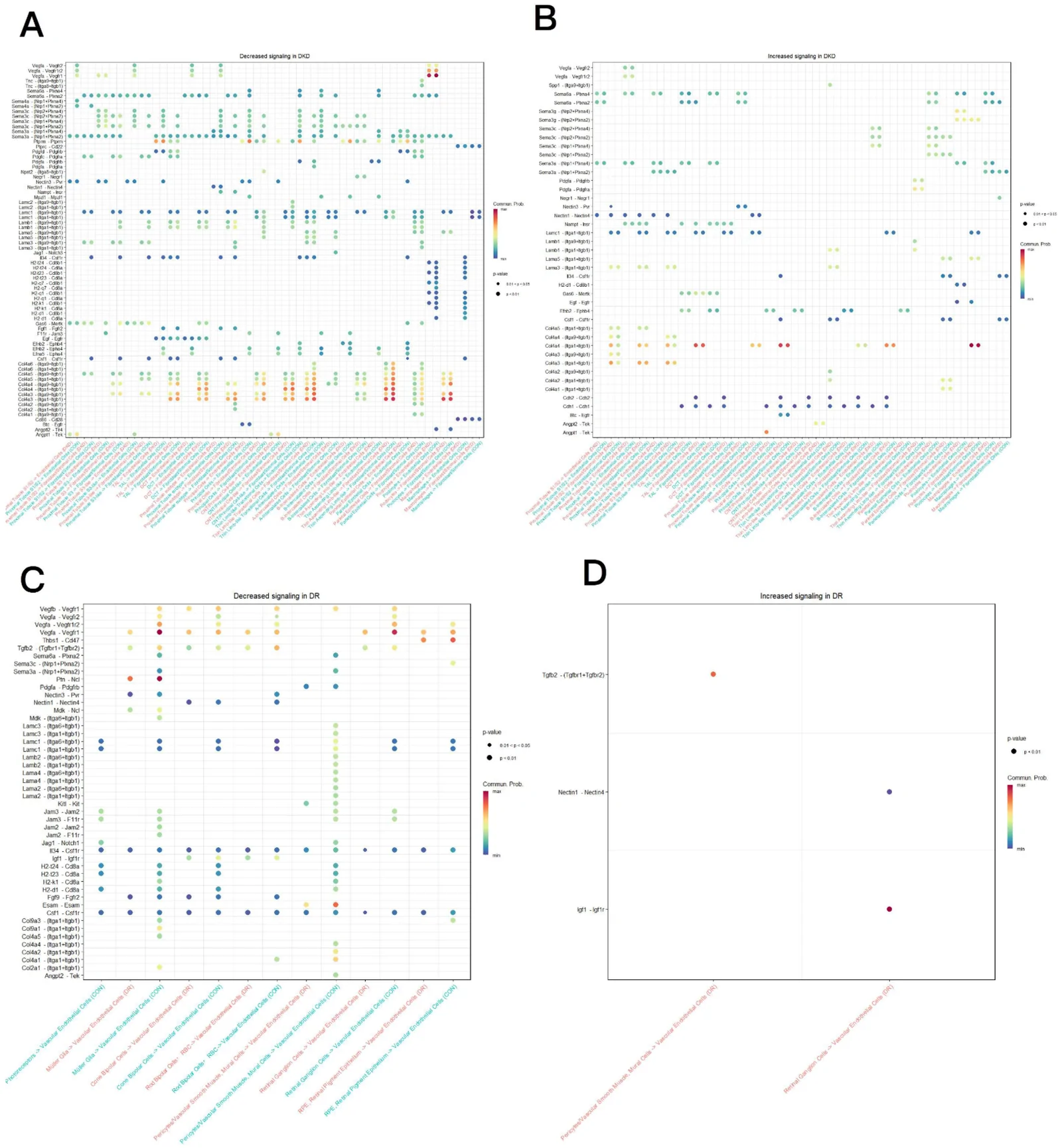
** Fibroblast-centric and endothelial-centric ligand-receptor analysis in DN. (A)** Downregulated ligand-receptor pairs where fibroblasts are receivers in DN. **(B)** Upregulated ligand-receptor pairs where fibroblasts are receivers in DN.

We further examined ligand-receptor pairs where fibroblasts (DN) or SMCs/endothelial cells (DR) act as receivers. In DR, pairs involving *Vegfb*, *Sema3*, *Lamc*, *Col9a3*, and *Col4a2* were downregulated, whereas pairs involving *Tgfb2*, *Nectin1*, and *Igf1* were upregulated (Figures 8E-F). In DN, pairs involving *Vegfa*, *Sema4*, *Lamc*, and *Col4a* showed significant changes (Figures 9A-B). The downregulation of ECM-related ligands (*Lamc*, *Col4a2*) may compromise basement membrane integrity, whereas upregulation of *Tgfb2* may promote fibrosis.

### Western Blotting Validated the Progressive Upregulation of COL1A2 Expression and Downregulation of APOLD1 Expression in a Diabetic Mouse Model

**Figure 10:**
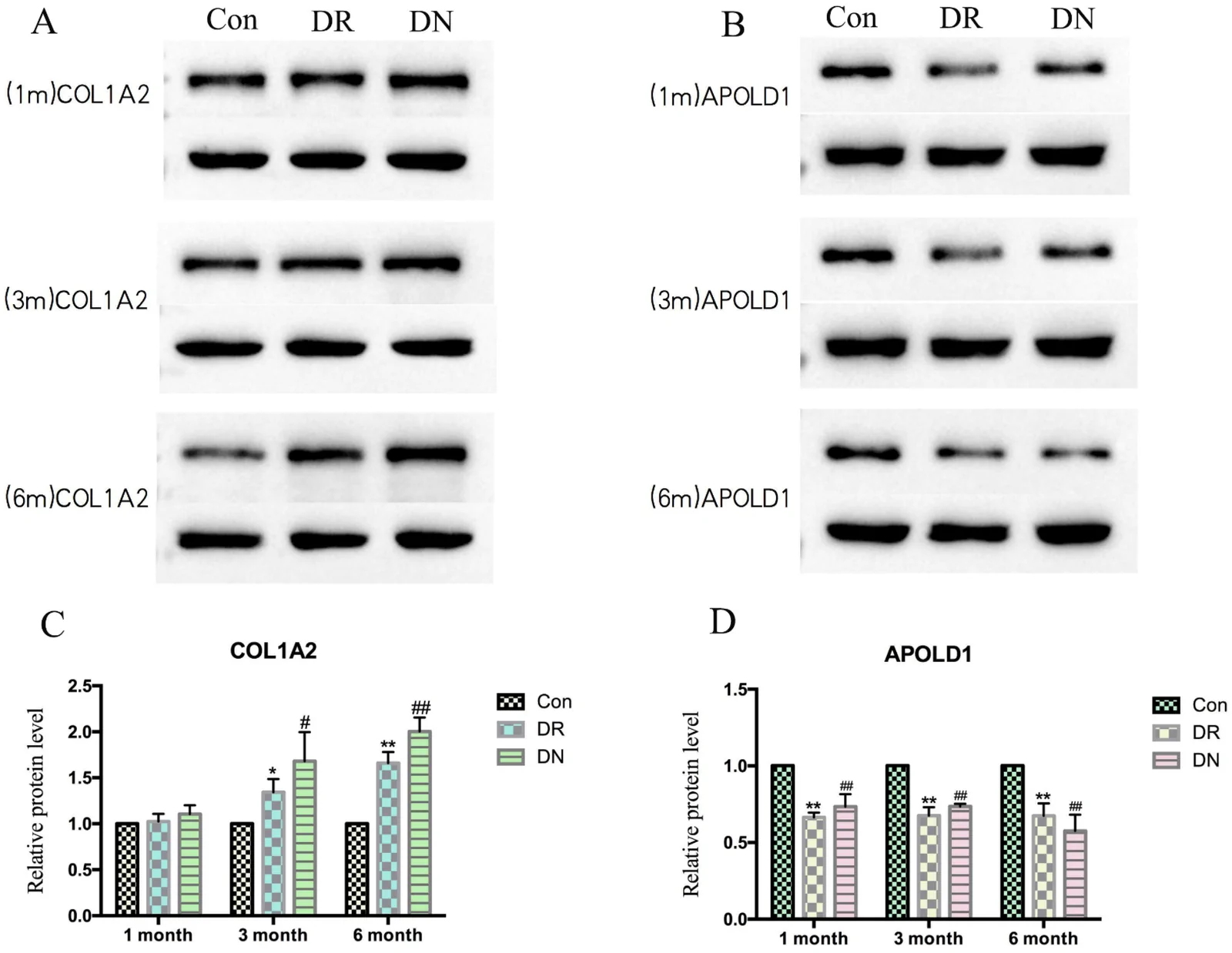
** Western blot validation of COL1A2 and APOLD1 protein expression in diabetic mouse model. (A)** Representative Western blot images of COL1A2 and APOLD1 in retinal tissues from control and db/db (T2D) mice at 1, 3, and 6 months. β-actin as loading control. **(B)** Representative Western blot images in renal tissues. **(C)** Quantitative analysis of COL1A2 protein levels normalized to β-actin (mean ± SEM, n=3 per group). **(D)** Quantitative analysis of APOLD1 protein levels normalized to β-actin. *p < 0.05, **p < 0.01 vs. control; #p < 0.05, ##p < 0.01 for comparisons between indicated time points within diabetic group (ANOVA with Tukey's test).

To experimentally validate our computational predictions, we performed Western blot analysis using retinal and renal tissues from db/db (T2D) mice at 1, 3, and 6 months post-diabetes onset. Consistent with the transcriptomic data, compared with control mice, in T2D model mice, COL1A2 protein levels progressively and significantly increased over time in both retinal and renal tissues (Figures 10A-C); conversely, APOLD1 protein expression significantly and progressively decreased over the same time course (Figures 10A-B, D). The temporal dynamics were consistent across the diabetic model, reinforcing upregulated COL1A2 expression and downregulated APOLD1 expression as core, conserved molecular events in diabetic microvascular complications. Notably, the changes were already detectable at 1 month (APOLD1 decrease) and became more pronounced at 3 and 6 months (COL1A2 increase), supporting the dual-axis model with early APOLD1 loss and late COL1A2 accumulation.

## Discussion

This integrative multiomics study systematically investigated the shared molecular architecture of diabetic retinopathy (DR) and diabetic nephropathy (DN), ultimately proposing a novel “dual-axis” mechanistic model supported by experimental validation. Through a comprehensive analysis of bulk transcriptomics, single-cell RNA sequencing, machine learning, and Western blotting, we identified COL1A2 as a consistently upregulated gene and APOLD1 as a consistently downregulated gene in both complications and elucidated their cell type-specific expression patterns, temporal dynamics, and dysregulated intercellular communication networks.

The identification of COL1A2 as a common upregulated hub gene reinforces the fundamental role of aberrant extracellular matrix remodeling and fibrosis in the terminal stages of both complications 14, 15. The upregulation of the expression of COL1A2, which encodes the α2 chain of type I collagen, by TGF-β1 and microRNAs such as miR-192 is a well-established driver of renal fibrosis in diabetic nephropathy 16, 17. Our Western blot data demonstrated time-dependent COL1A2 protein accumulation in both retinal and renal tissues in db/db diabetic mice, reflecting the progression of structural damage. This finding, coupled with pseudotime trajectory analysis showing peak expression in late disease-associated cell trajectories (fibroblasts in DN and pericytes in DR), which revealed a late structural remodeling axis driven by upregulated COL1A2 expression. Herein, 'late' refers to the relatively late phase within the current animal model's disease course (6 months) and the later stages of the pseudotime trajectories, rather than clinical end stage renal fibrosis or proliferative DR in humans. Notably, pseudotime analysis revealed that COL1A2 expression increased in late-stage disease-associated cell states characterized by the high expression of Acta2, Tgfb1, and Fn1, further supporting its association with end-stage fibrotic remodeling 18-20. Mechanistically, COL1A2 may contribute to fibrosis not only by increasing ECM deposition but also by activating integrin-mediated signaling pathways (PI3K-Akt) that promote cell survival and myofibroblast differentiation.

The parallel finding that APOLD1 is a common downregulated gene is particularly striking. Although the functional role of APOLD1 in diabetic complications is less established than that of COL1A2, its consistent downregulation across both tissues and both diabetic models was confirmed by Western blot analyses, suggesting that APOLD1 may act as a factor linked to early microvascular dysfunction 21, 22. APOLD1 is known to be enriched in endothelial cells and has been implicated in angiogenesis and vascular barrier function 23. Notably, our scRNA-seq data show that APOLD1 is predominantly expressed in fibroblasts rather than endothelial cells in DN, which appears to differ from previous reports characterizing APOLD1 as a vascular endothelial gene 24. This discrepancy may reflect species differences (mouse models vs. human tissues), cellular reprogramming in the diabetic microenvironment, or the specific disease stage analyzed. Furthermore, it is possible that APOLD1 expression is not strictly cell-type-restricted and may be induced in fibroblast populations under pathological conditions. Further studies are warranted to clarify this cell-type specificity and its functional implications in diabetic complications. Our data show that APOLD1 expression is induced early (evident at 1 month) and progresses gradually, preceding the significant increase in COL1A2 expression. This expression pattern, combined with pseudotime analysis showing that APOLD1 expression peaks in early- or mid-stage disease trajectories, positions it within the “early dysfunction axis”. The downregulation of APOLD1 expression may represent an early event that renders the microvasculature susceptible to subsequent inflammation and fibrosis 25. Clinically, early (non-proliferative) DR is often asymptomatic, and patients may miss the therapeutic window. The early change in APOLD1 (1 month in the animal model, corresponding to the early metabolic stress stage in humans) suggests that APOLD1 may help identify at-risk patients before visible fundus changes occur, potentially enabling earlier intervention. APOLD1 loss may compromise endothelial tight junctions and Weibel-Palade body function, leading to increased vascular permeability and leukocyte adhesion—early steps in diabetic microangiopathy. However, direct evidence for these mechanisms in the context of diabetic complications is currently lacking, and this remains a hypothesis to be tested in future studies.

The cell type-specific expression patterns revealed by single-cell RNA sequencing provide insights into cellular pathogenesis. The localization of these genes in kidney fibroblasts and retinal pericytes highlights the shared vulnerability of the vascular-supporting cell types across different tissues, albeit with subtle differences in the principal cellular protagonists. Pericyte dysfunction is a hallmark of DR, leading to the breakdown of the blood-retinal barrier; fibroblast activation and matrix production are central to DN 26, 27. Our data suggest that these distinct target cells may exhibit similar molecular response programs involving COL1A2 and APOLD1. Notably, in DN tissues, both genes were specifically coexpressed in fibroblast subsets, whereas in DR, their expression was predominantly localized to pericytes/vascular smooth muscle cells, although APOLD1 was also clearly expressed in endothelial cells 28, 29. This divergence may reflect the different cellular compositions of the retinal and renal microvasculature: the retina has a high pericyte-to-endothelial ratio and tight pericyte coverage, whereas the kidney interstitium is rich in fibroblasts that respond to injury with matrix deposition. Thus, the primary cell types that respond to metabolic stress may differ, yet both converge on common molecular pathways involving APOLD1 and COL1A2. The late high expression of COL1A2 in pericytes is of particular clinical relevance. Pericyte dysfunction and loss are key events leading to blood-retinal barrier breakdown and microaneurysm formation—hallmark pathological changes in DR. The temporal accumulation of COL1A2 in pericytes may therefore reflect progressive pericyte injury and contribute to the structural deterioration of the retinal microvasculature.

Furthermore, our systems-level intercellular communication analysis revealed the extensive reprogramming of cell-cell signaling networks in both complications but with striking differences in direction. In DN tissues, immune-inflammatory pathways such as SPP1, RANKL, and CD45 were activated, and the WNT and NOTCH pathways were suppressed 30. These findings suggest that in DN, the microenvironment is dominated by immune activation and regenerative signal suppression, favoring chronic inflammation and fibrosis. In contrast, in DR tissues, the VEGF, LAMININ, and EDN pathways are suppressed 31, 32. In contrast, in DR tissues, the VEGF, LAMININ, and EDN pathways were suppressed in our analysis. Although VEGF is well-established as a key mediator in proliferative DR, its expression is not consistently elevated in non-proliferative DR. Haghjooy Javanmard et al. reported that aqueous VEGF levels in NPDR patients did not differ significantly from non-diabetic controls, and suggested that decreased sVEGFR-1 may represent an initial compensatory event in early NPDR 33. Furthermore, Yang et al. demonstrated that VEGF-induced angiogenesis is a critical compensatory response to microvascular rarefaction in the diabetic retina 34. The downregulation of VEGF pathway activity observed in our DR samples may therefore reflect the early (non-proliferative) stage of retinopathy in the datasets analyzed, or a compensatory response following initial hyperglycemic insult.

The downregulation of laminin expression may compromise basement membrane stability, contributing to vascular leakage. The suppression of WNT and NOTCH signaling in diabetic kidney tissue suggests a potential loss of regulatory signals 35, 36. The alterations in VEGF and ECM-related signals (LAMININ and COL4A) in both tissues highlight the complex interplay between growth factors and the matrix in the diabetic microenvironment 37, 38. These dysregulated networks may provide the context in which dual-axis molecular changes occur. For example, early APOLD1 downregulation may be triggered by the loss of VEGF or other endothelial survival signals, whereas late COL1A2 upregulation may be driven by TGF-β and mechanical stress from altered ECM. The late high expression of COL1A2 in pericytes is of particular clinical relevance. Pericyte dysfunction and loss are key events leading to blood-retinal barrier breakdown and microaneurysm formation—hallmark pathological changes in DR. The temporal accumulation of COL1A2 in pericytes may therefore reflect progressive pericyte injury and contribute to the structural deterioration of the retinal microvasculature.

Single-cell analyses after *in silico* COL1A2 knockout provided additional observations. The perturbed response in DN fibroblasts is predominantly associated with immune-related genes, whereas vascular and stress/metabolic responses are more prominent in DR SMCs 39, 40. This differential response pattern suggests that even the same gene may be linked to distinct molecular programs in different tissue microenvironments — in the kidney, COL1A2 may be more involved in immune-stromal crosstalk; in the retina, COL1A2 may be more involved in vascular stress responses 41, 42. These findings provide candidate directions for subsequent experimental validation focusing on ECM-receptor interactions and adhesion-inflammation coupling mechanisms 43.

Finally, the development of machine learning models using comorbidity-derived gene signatures demonstrates the translational potential of our findings 44. By constructing 120 combinatorial machine learning models and achieving relatively good validation AUCs in the DR internal hold-out test set and the DN independent external validation set, we identified gene signatures that may help identify patients at high risk for concurrent microvascular complications 45. The small gap between cross-validation and the corresponding validation AUCs suggests stable predictive performance, although the DR model requires evaluation in a truly independent external dataset to establish generalizability. Therefore, comorbidity-derived gene signatures remain potentially useful predictors. The fact that these signatures were derived from a minimal set of genes (intersection of module genes and DEGs) enhances their clinical applicability, as they can be measured by targeted assays such as qPCR or multiplex protein assays. These models could inform future risk stratification approaches. Such gene signatures could be integrated as a molecular complement to existing fundus photography plus artificial intelligence screening pipelines, adding biological information to image-based assessment for risk stratification. This combined strategy may be especially valuable for patients with early-stage diabetes who have no clinically apparent retinopathy but exhibit severe metabolic abnormalities, potentially enabling more refined risk stratification and closer surveillance.

From a translational perspective, the feasibility of detecting these markers in clinically accessible samples deserves consideration. COL1A2 is a secreted collagen and may theoretically be detectable in peripheral blood, tear fluid, or aqueous humour; in contrast, APOLD1 is a membrane-associated protein and may have limited detectability in body fluids. Future studies should verify the detectability of these markers in clinically accessible samples and evaluate their correlation with retinopathy severity, which would be a prerequisite for their clinical application as non-invasive biomarkers.

Several limitations of this study should be acknowledged. First, although we provide computational evidence and initial protein-level validation, the findings are correlational rather than causal. *In vitro* APOLD1 and COL1A2 knockdown/overexpression experiments in relevant cell types (fibroblasts, pericytes, and endothelial cells) and *in vivo* studies using appropriate models are needed to establish functional roles. *In silico* knockout analysis generated hypotheses — for example, the immune-related perturbation response in DN fibroblasts warrants investigating the potential involvement of COL1A2 in local immune interactions. Second, while we used multiple public datasets to enhance robustness, heterogeneity among datasets (e.g., different platforms and disease stages) remains a potential confounder. Third, our animal models, although covering T2D, may not fully capture the complexity of human disease. Specifically, the 6-month db/db mouse model represents the relatively late phase within this model's disease course, rather than clinical end-stage renal fibrosis or proliferative DR in humans. Fourth, the upstream regulatory networks controlling COL1A2 and APOLD1 expression remain to be elucidated. Potential regulators such as transcription factors (e.g., TGF-β/SMAD for COL1A2) and epigenetic modifiers should be explored in future studies.

## Conclusions

Through integrative bulk and single-cell transcriptomics, machine learning, network biology, and experimental validation, we delineated a molecular framework for diabetic retinopathy and nephropathy comorbidity. We propose that the coordinated downregulation of APOLD1 expression (early dysfunction axis) and upregulation of COL1A2 expression (late structural axis) occur within the context of dysregulated intercellular communication and are associated with concurrent microvascular disease. The validation of these dynamics at the protein level in a diabetic mouse model, the cell type-specific localization revealed by single-cell RNA sequencing, and the temporal patterns revealed by pseudotime analysis support the biological relevance of our findings. These core genes and pathways represent promising candidates for early detection, risk stratification, and therapeutic interventions in diabetic renal-retinal syndrome and warrant further functional and clinical investigation.

## Supplementary Material

Supplementary figures.
