# Supplementary material for: COL1A2 and APOLD1 Define a Dual-Axis Molecular Framework for Diabetic Nephropathy-Retinopathy Comorbidity: An Integrative Multiomics and Machine Learning Study in Mouse Models
Source: Int J Med Sci. 2026 Aug 11;23(9):3044–65. doi: 10.7150/ijms.136651 (PMC13539923; doi:10.7150/ijms.136651)
Supplement: Supplementary file 1 [file ijmsv23p3044s1.pdf]

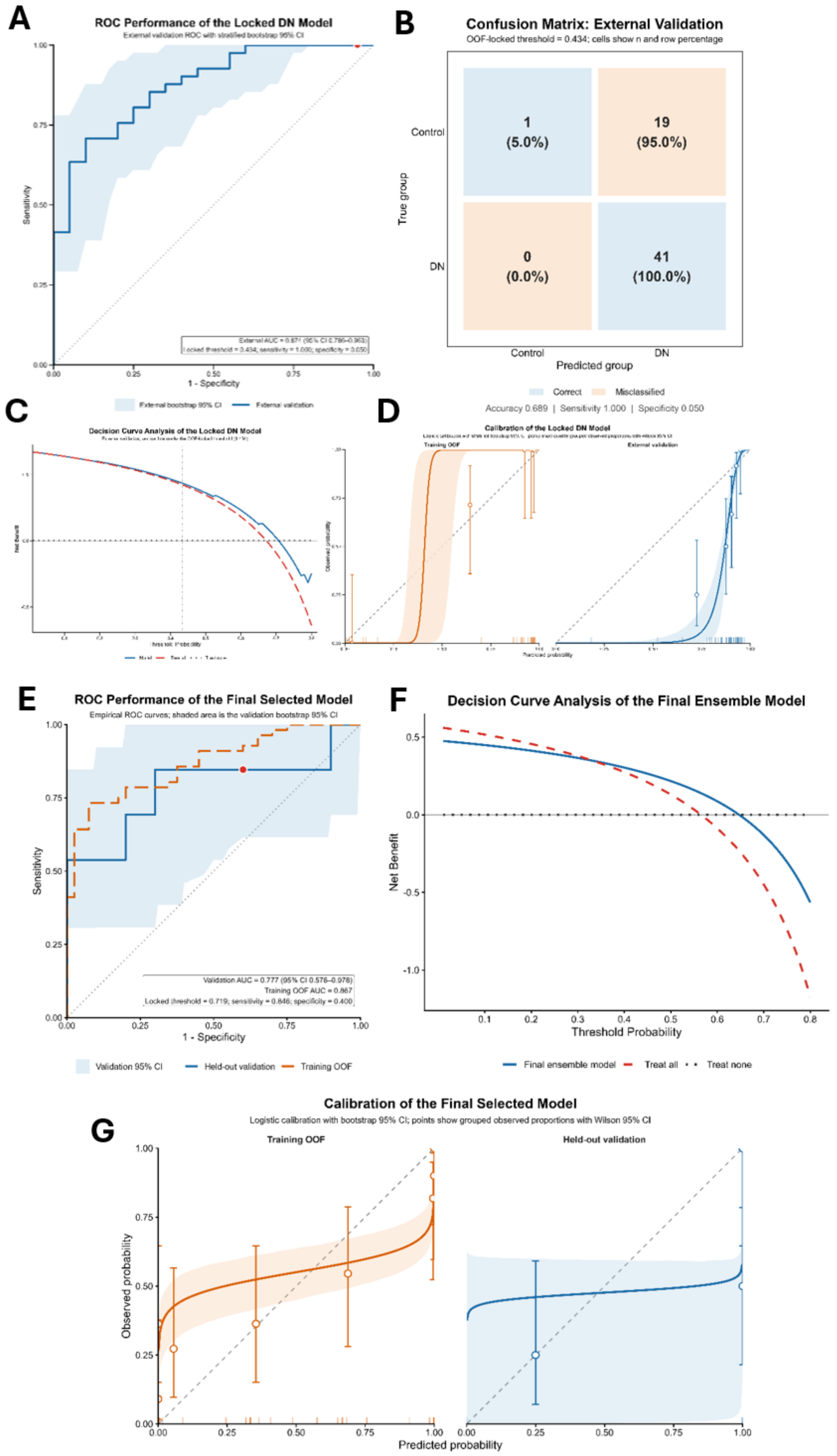

Figure S1. Discrimination, clinical net benefit, and calibration of the final DR&DN ensemble model.

The training cohort comprised 36 samples, including 27 patients with DN and 9 controls, while the independent external validation cohort comprised 61 samples, including 41 patients with DN and 20 controls. Sample-level out-of-fold predictions were generated

using five-fold outer cross-validation. The radial basis function support vector machine model (svmRad) was locked using training OOF information only, without access to external labels or external AUC. (A)ROC curve of the locked model in the external validation cohort. The blue step curve represents the empirical ROC curve, and the light-blue shaded region represents the stratified bootstrap 95% confidence interval. The external validation AUC was 0.874 (95% CI, 0.786–0.963). The red point indicates the threshold of 0.434, which was determined from the training OOF predictions using the Youden index. At this threshold, external sensitivity was 1.000 and specificity was 0.050. The grey dotted diagonal indicates random classification. (B)Confusion matrix of the locked DN classification model in the external validation cohort. (C)Decision curve analysis in the external validation cohort. The solid blue line represents the model-based strategy, the dashed red line represents the treat-all strategy, and the black dotted line represents the treat-none strategy. The vertical grey dot-dashed line indicates the training OOF-locked threshold of 0.434. The model provided greater net benefit than both default strategies across an approximate threshold-probability range of 0.19–0.70.(D)Calibration of the locked model. Training OOF calibration is shown on the left and external validation calibration on the right. Solid curves represent logistic calibration functions, and shaded regions represent stratified bootstrap 95% confidence intervals. Open circles indicate quintile-grouped observed DN proportions, with error bars representing Wilson 95% confidence intervals. The grey dashed diagonal represents perfect calibration.(E) Receiver operating characteristic (ROC) curves of the final selected model. The orange dashed line represents the out-of-fold (OOF) ROC curve in the training set, and the blue solid line represents the ROC curve in the internal hold-out validation set. The light-blue shaded area indicates the bootstrap 95% confidence interval for the validation ROC curve. The training OOF AUC was 0.867, whereas the internal hold-out validation AUC was 0.777 (95% CI, 0.576–0.978). The red point denotes the operating point at the prespecified locked threshold of 0.719, corresponding to a sensitivity of 0.846 and a specificity of 0.400.(F) Decision curve analysis of the final ensemble model. The blue solid line represents the final ensemble model, the red dashed line represents the treat-all strategy, and the black dotted line represents the treat-none strategy.(G) Calibration plots of the final selected

model. The left and right panels show the training OOF and internal hold-out validation results, respectively. Solid curves represent logistic calibration estimates, shaded areas indicate bootstrap 95% confidence intervals, and circles with error bars denote grouped observed proportions with Wilson 95% confidence intervals. The gray dashed line represents perfect calibration, and rug marks show the distribution of predicted probabilities.

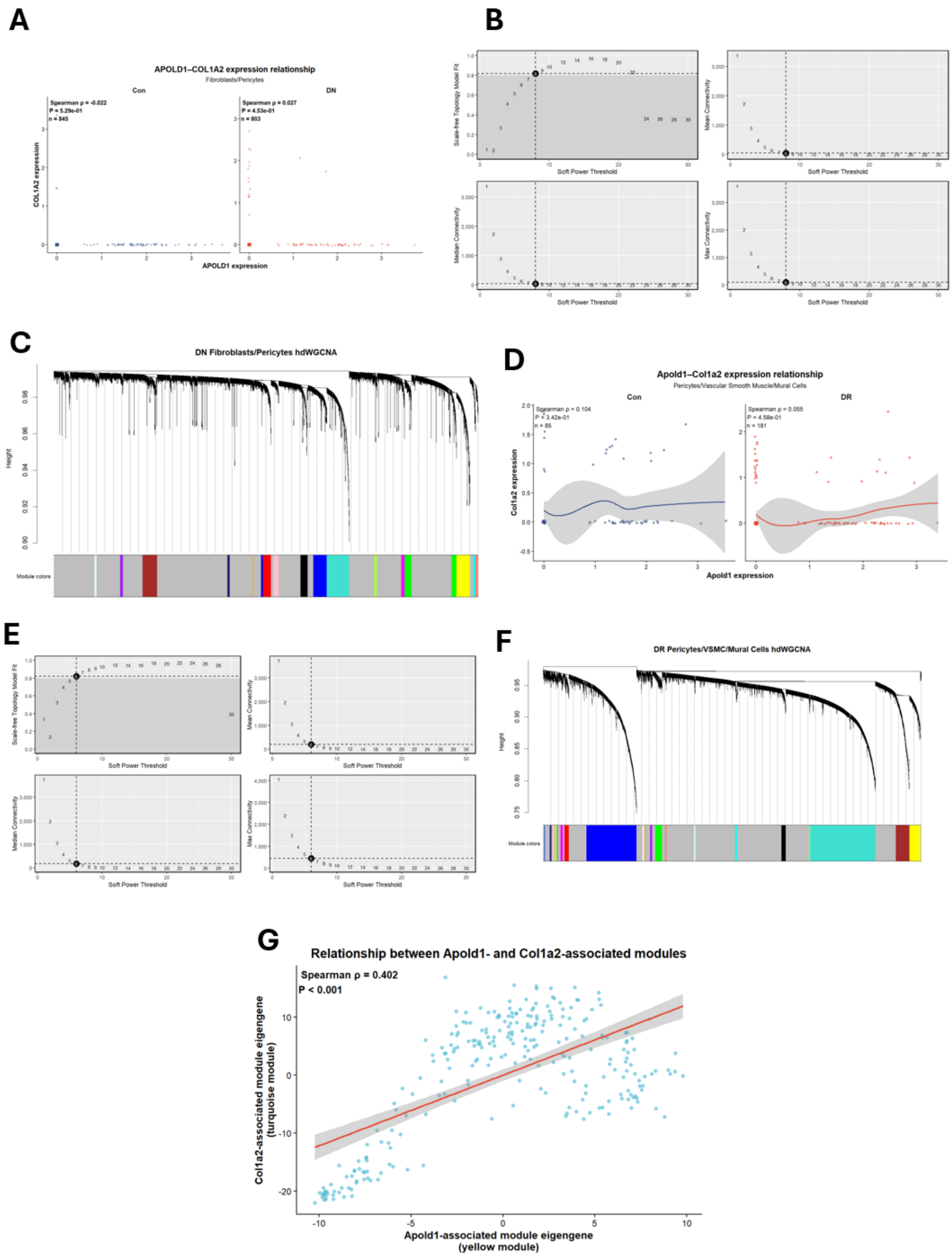

Figure S2. Spearman correlation and hdWGCNA co-expression network analysis of APOLD1 and COL1A2 in DN and DR single-cell datasets.

(A) Spearman correlation analysis of APOLD1 and COL1A2 expression in Fibroblasts/Pericytes from the diabetic nephropathy (DN) dataset. No significant correlation was observed in either the control group (Con; Spearman ρ = −0.022, P = 0.529,

n = 845) or the DN group (Spearman $\rho = 0.027$, $P = 0.453$, n = 803).(B) Selection of the soft-thresholding power for hdWGCNA network construction in DN Fibroblasts/Pericytes. A soft-thresholding power of 8 was selected based on the scale-free topology model fit together with mean, median, and maximum connectivity characteristics.(C) Hierarchical clustering dendrogram and module assignment generated by hdWGCNA in DN Fibroblasts/Pericytes. Different colors represent distinct co-expression modules identified by dynamic tree cutting, whereas the grey category represents genes that were not robustly assigned to a specific co-expression module.(D) Spearman correlation analysis of Apold1 and Col1a2 expression in Pericytes/Vascular Smooth Muscle/Mural Cells from the diabetic retinopathy (DR) dataset. No significant correlation was detected in either the control group (Con; Spearman $\rho = 0.104$, $P = 0.342$, n = 85) or the DR group (Spearman $\rho = 0.055$, $P = 0.458$, n = 181).(E) Selection of the soft-thresholding power for hdWGCNA network construction in DR Pericytes/Vascular Smooth Muscle/Mural Cells. A soft-thresholding power of 6 was selected according to the scale-free topology fit and network connectivity characteristics.(F) Hierarchical clustering dendrogram and module identification by hdWGCNA in DR Pericytes/Vascular Smooth Muscle/Mural Cells. Different colors indicate distinct co-expression modules.(G) Correlation between the Apold1-associated yellow module and the Col1a2-associated turquoise module. hdWGCNA assigned Apold1 and Col1a2 to distinct co-expression modules, whereas the corresponding module eigengenes showed a moderate positive correlation (Spearman $\rho = 0.402$, $P < 0.001$).
